# Severe allergic symptoms to peach are a risk factor for severe symptoms to other plant food allergens

**DOI:** 10.1186/2045-7022-1-S1-P79

**Published:** 2011-08-12

**Authors:** Ambra Mascheri, Joseph Scibilia, Laura Farioli, Chrysi Stafylaraki, Valerio Pravettoni, Marta Piantanida, Laura Primavesi, Corrado Mirone, Michele Nichelatti, Alessandro Marocchi, Elide Anna Pastorello

**Affiliations:** 1 Niguarda Cà Granda Hospital, Allergology and Immunology Unit, Milan, Italy; 2 Niguarda Cà Granda Hospital, Department of Laboratory Medicine, Milan, Italy; 3 Foundation IRCCS Ca’ Granda Ospedale Maggiore Policlinico, Clinical Allergy and Immunology Unit, Milan, Italy; 4 Niguarda Cà Granda Hospital, Center for Clinical Research, Milan, Italy

## Background

The hypersensitivity reactions to plant-food allergens may consist of oral allergic symptoms (OAS) only or OAS plus systemic symptoms up to anaphylaxis. Currently there is great interest in evaluating the risk factors for developing the more severe and systemic reactions.

## Objective

A group of 148 peach allergic patients, recruited in a ongoing clinical study (Clinical Trials.gov, protocol ID NCT00715156) aimed at studying the relationship between the clinical manifestations to peach and the positivity to different peach major allergens (Pru p 3, 1 and 4), were divided into 2 groups according to peach induced symptoms: group A (mild-OAS; 76 pts) and group B (severe-OAS with systemic symptoms, 72 pts). These patients were examined to see if severe allergic symptoms to peach were a risk factor for developing severe symptoms to other plant foods allergens.

## Methods

We investigated the type of reaction to peach and to other foods by means of a clinical questionnaire and confirmed their allergy with open food challenge (fresh fruits and vegetables) or double blind challenge (maize, rice and wheat) and, when necessary, double blind challenge + exercise. In all the patients we performed skin prick test, prick-prick with natural food, and IgE-specific levels for all the considered plant-food allergens and for the following recombinant allergens Pru p 1, 3, 4, Bet v 1, 2 and 4. Then we compared all the reported parameter between the two groups.

## Results

In general there was a significant association between patients belonging to group B (severe peach induced symptoms-OAS) and having severe symptoms to many of the other tested plant food allergens. Moreover for the majority of these foods, the IgE positivity to rPru p 3 resulted to be a biological marker of food allergy severity.

## Conclusions

This study suggests that patients with severe allergic symptoms to peach have a major risk of presenting severe allergic symptoms to other plant-food allergens.

